# Canagliflozin Ameliorates Oxidative Stress and Autistic-like Features in Valproic-Acid-Induced Autism in Rats: Comparison with Aripiprazole Action

**DOI:** 10.3390/ph16050769

**Published:** 2023-05-19

**Authors:** Mohammed Moutaz Nakhal, Petrilla Jayaprakash, Salahdein Aburuz, Bassem Sadek, Amal Akour

**Affiliations:** 1 Department of Biochemistry and Molecular Biology Sciences, College of Medicine and Health Sciences, United Arab Emirates University, Al Ain P.O. Box 15551, United Arab Emirates; 2 Department of Pharmacology & Therapeutics, College of Medicine and Health Sciences, United Arab Emirates University, Al Ain P.O. Box 15551, United Arab Emiratessaburuz@uaeu.ac.ae (S.A.); 3 Zayed Center for Health Sciences, United Arab Emirates University, Al-Ain P.O. Box 17666, United Arab Emirates; 4 Department of Biopharmaceutics and Clinical Pharmacy, School of Pharmacy, The University of Jordan, Amman 11942, Jordan

**Keywords:** autism spectrum disorder, SGLT2 inhibitors, canagliflozin, aripiprazole, behavioral assessments, biochemical assays, VPA-induced ASD, oxidative stress biomarkers, rats

## Abstract

Based on their proven anti-inflammatory and antioxidant effects, recent studies have examined the therapeutic potential of the sodium-glucose cotransporter 2 (SGLT2) inhibitors in neurodevelopmental disorders such as autism spectrum disorder (ASD). Therefore, the aim of this study is to assess the effects of subchronic systemic treatment with intraperitoneal (i.p.) canagliflozin (20, 50, and 100 mg/kg) compared to aripiprazole (ARP) (3 mg/g, i.p.) in a valproic acid (VPA)-induced rat model of autism. The behavioral characteristics of ASD, oxidative stress, and acetylcholinesterase (AChE) activity in rats with ASD-like behaviors, which were induced by prenatal exposure to VPA, were evaluated. The behavioral assessment methods used for this study were the open field test (OFT), the marble-burying test (MBT), and the nestlet-shredding test (NST) to examine their exploratory, anxiety, and compulsiveness-like actions, while the biochemical assessment used for this study was an ELISA colorimetric assay to measure ASD biomarker activity in the hippocampus, prefrontal cortex, and cerebellum. Rats that were pretreated with 100 mg/kg of canagliflozin displayed a significantly lower percentage of shredding (1.12 ± 0.6%, *p* < 0.01) compared to the ARP group (3.52 ± 1.6%). Pretreatment with (20 mg/kg, 50 mg/kg, and 100 mg/kg) canagliflozin reversed anxiety levels and hyperactivity and reduced hyper-locomotor activity significantly (161 ± 34.9 s, *p* < 0.05; 154 ± 44.7 s, *p* < 0.05; 147 ± 33.6 s, *p* < 0.05) when compared with the VPA group (303 ± 140 s). Moreover, canagliflozin and ARP mitigated oxidative stress status by restoring levels of glutathione (GSH) and catalase (CAT) and increasing the levels of malondialdehyde (MDA) in all tested brain regions. The observed results propose repurposing of canagliflozin in the therapeutic management of ASD. However, further investigations are still required to verify the clinical relevance of canagliflozin in ASD.

## 1. Introduction

Autism spectrum disorder (ASD) is a neurodevelopmental disorder with an increasing incidence rate, characterized by repetitive behaviors, learning difficulties, and deficits in social communication and interactions [1]. It has been reported that around 1% of the population have been diagnosed with the common neurodevelopmental disorder known as autism spectrum disorder (ASD) [1]. The global prevalence of ASD has reached one in 161 people, [2].

Presently, there are no definitive medications to cure ASD, hence most therapeutic strategies, including pharmacological agents such as aripiprazole (ARP), dietary therapies, and behavioral interventions aim at ameliorating ASD symptoms, improving children’s social interactions, and assisting their learning process [3].

The importance of diets known to be antioxidant or anti-inflammatory in neurological diseases lies in targeting specific proteins in the brain that potentially could lead to neuroprotective effects, which was noticeable in dietary resveratrol when consumed by transgenic Alzheimer’s disease (AD) mouse models through increasing glycogen synthase kinase 3-b phosphorylation, drebrin, and transthyretin levels in the brain [4]. In addition, supplementation with multi-vitamins, vitamin D and minerals such as zinc, magnesium, and selenium achieve synergistic effects when used in combination in ASD [5].

The etiology of ASD is complicated, and it has been linked with genetic factors or environmental elements, such as infections, toxins, or several medications, which cause several epigenetic changes [6]. Additionally, the risk of developing drug-induced ASD is increased during the second trimester of fetal development [7]. Accordingly, medications such as antipsychotics, anticonvulsants, and antidepressants have the potential of inducing ASD during pregnancy, e.g., the antiepileptic drug valproic acid (VPA), which is prescribed mainly in bipolar disorder and epilepsy [8]. In the present study, ASD was induced in accordance with a systematic review that has stated that drug-induced ASD is obtained by prenatal systemic subchronic exposure to a dose of 500 mg/kg of VPA injected intraperitoneally (i.p.) at 12.5 gestational days in Wistar rats [9].

Sodium-glucose cotransporter 2 (SGLT2) inhibitors are recognized as oral glucose-lowering medications that operate by reducing the reabsorption of the renal glucose [10]. Examples of these drugs include dapagliflozin, ertugliflozin, empagliflozin, canagliflozin, ipragliflozin, tofogliflozin, luseogliflozin, and remogliflozin [11]. Additionally, and according to the American Diabetes Association (ADA), SGLT2 inhibitors are the first-line agents for pharmacological intervention in the treatment of type 2 diabetes mellitus (T2DM), especially in patients who are diagnosed with heart failure (HF) [12,13]. Moreover, according to the European Society of Cardiology (ESC), the SGLT2 inhibitors are prescribed as an independent treatment of HF with a reduced ejection fraction, regardless of the diabetic status [12,13]. Notably, SGLT2 inhibitors have a variety of pleiotropic benefits, such as improving the visceral adiposity, reduction of body weight, lowering blood pressure, anti-inflammatory effect, oxidative stress downregulation, and normalizing serum uric acid levels as well as ameliorating lipid profile [14]. Interestingly, SGLT2 inhibitors such as canagliflozin and empagliflozin have shown promising results in the field of neurological disorders, including Alzheimer’s disease, Parkinson’s disease, and epilepsy [15], as they possess the advantage of crossing the blood–brain barrier (BBB), after which they have shown ability to reduce oxidative stress and inflammatory processes, which validates their activation, absorbance, and exposure profiles in the brain [16,17,18,19,20,21,22,23,24,25,26,27,28,29]. In addition, canagliflozin has been found to exhibit a dual activity on both SGLT1 and SGLT2 receptors, which are distributed in various brain areas of mammalian brain, such as the hippocampus, prefrontal cortex, and cerebellum [16].

Since there is a lack of data on SGLT2 inhibitor efficacy in ASD, and based on this scientific gap particularly, it is tempting to speculate that canagliflozin could be utilized in neurological disorders such as ASD. Therefore, the current series of experiments was aimed to further examine the effects of subchronic systemic treatment with canagliflozin on the core ASD-like behavioral characteristics, oxidative stress levels, and acetylcholinesterase (AChE) activity in VPA-exposed rats in comparison with ARP, which were evaluated by applying a marble-burying test (MBT), nestlet-shredding test (NST), and open field test (OFT). Moreover, the effects of canagliflozin on several oxidative stress markers and AChE activity were assessed in the hippocampus, prefrontal cortex, and cerebellum, as these specific brain regions are involved in executive and cognitive functions and demonstrate exaggerated oxidative stress associated with ASD-like social behavioral deficits in rodents [30].

## 2. Results

### 2.1. Effects of Canagliflozin on Rats ASD-like Anxiety and Exploratory Behaviors

#### 2.1.1. Open Field Test (OFT)

The OFT is a task used to evaluate locomotor activity as well as fear- and anxiety-like behaviors in experimental rodents [31]. The results observed in OFT were utilized to evaluate the effects of subchronic systemic administration of canagliflozin (20–100 mg/kg, i.p.) and the reference drug aripiprazole (ARP, 3 mg/kg, i.p.) and are illustrated in Table 1. Statistical analyses of results observed in the OFT showed that VPA-exposed animals pretreated with normal saline (10 mL/kg, i.p.) spent noticeably less time in the center (3.00 ± 0.894 s, *p* < 0.01) when compared with the control group (14.83 ± 3.188 s) and the canagliflozin (100 mg/kg, i.p.) group (13.00 ± 3.09 s). However, VPA-exposed animals pretreated with the reference drug ARP, canagliflozin (50 mg/kg, i.p.), and canagliflozin (100 mg/kg, i.p.) groups spent significantly more time in the center than the saline-treated VPA-exposed group, with 9.33 ± 4.41 s, 7.83 ± 1.72 s, 13.00 ± 3.09 s, respectively (all *p* < 0.05) compared to 3.00 ± 0.89. Moreover, the results revealed that all canagliflozin treatment significantly reduced the increased levels of locomotor activity of tested animals when compared with the saline-treated VPA-exposed control group (*p* < 0.05). Additionally, systemic pretreatment with canagliflozin (100 mg/kg, i.p.) significantly decreased aggressive-like self-grooming behaviors of VPA-exposed rats, with 5.50 ± 0.83 (*p* < 0.05) and 9.50 ± 2.88 for canagliflozin-treated and VPA-exposed rats, respectively. However, lower doses of canagliflozin failed to mitigate aggressive-like self-grooming behaviors of tested animals. Moreover, the results observed in the OFT showed that VPA-exposed rats displayed higher anxiety-like behaviors accompanied with lower levels of exploratory behaviors compared with control group. However, canagliflozin was able to ameliorate the ASD-like behaviors in a dose-dependent manner (20, 50, 100 mg/kg). The positive control group treated with 3 mg/kg of aripiprazole had significantly less locomotion than the VPA-exposed group.

In addition, defecation frequencies were significantly lower in the saline-exposed group (3.33 ± 0.81, *p* < 0.05) when compared to the VPA-exposed group (6.00 ± 1.78).

#### 2.1.2. Marble-Burying Test (MBT)

**Figure 1:**
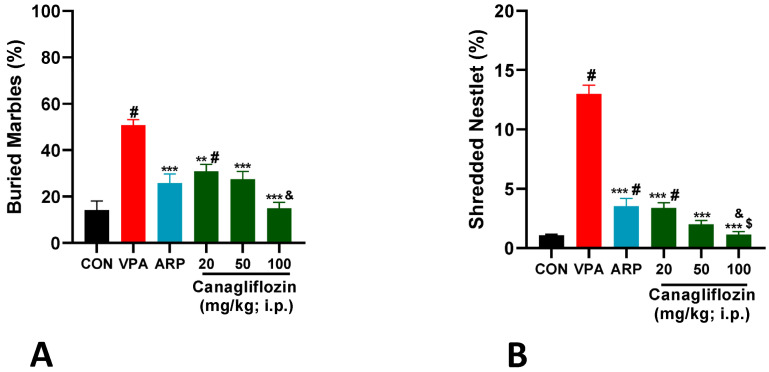
(**A**) Canagliflozin and aripiprazole mitigated stereotyped repetitive behavior in the marble-burying test (MBT). Marbles were counted after a 30 min testing session applying the same treatments. VPA-induced ASD rats treated with saline (VPA) displayed significantly increased repetitive behaviors when compared to control rats (CON) and all treatment groups. Canagliflozin (20, 50, and 100 mg/kg, i.p.) or the reference drug aripiprazole (ARP, 3 mg/kg, i.p.) was injected subchronically for 29 days in VPA-exposed rats. Values are expressed as mean ± SEM (*n* = 6). (**B**) Canagliflozin and aripiprazole attenuated increased obsessive–compulsive features in the nestlet-shredding test (NST). Repetitive nestlet-shredding behavior was measured after a 30 min testing session. VPA-exposed rats (VPA) demonstrated significantly elevated stereotyped repetitive behaviors compared to saline-exposed rats (CON). Canagliflozin (20, 50, and 100 mg/kg) or the reference drug aripiprazole (ARP, 3 mg/kg, i.p.) was administered subchronically for 29 days. The administration of aripiprazole (ARP, 3 mg/kg, i.p.) or canagliflozin (20, 50, or 100 mg/kg, i.p.) attenuated stereotyped repetitive behavior of VPA-exposed rats. Values are expressed as ± SEM (*n* = 6). # *p* < 0.05 when compared with the CON group, & *p* < 0.05 when compared with the canagliflozin 20 mg group, $ *p* < 0.05 when compared with the ARP group, ** *p* < 0.01 when compared with the VPA group, *** *p* < 0.001 when compared with the VPA group.

A marble-burying paradigm was used to assess the effects of canagliflozin and the reference drug ARP on the stereotyped and compulsive-like behaviors of VPA-exposed and control rats, and previously applied experimental procedures were used in the current series of experiments [32]. The observed results showed that VPA-exposed animals displayed a significantly increased percentage of buried marbles (50.83 ± 5.8%, *p* < 0.001) when compared with the control group (14.16 ± 9.7%) (Figure 1A). However, all treatment groups including ARP (25.8 ± 9.7%, *p* < 0.001), canagliflozin (20 mg, 30.8 ± 7.3%, *p* < 0.01), canagliflozin (50 mg, 27.50 ± 8.2%, *p* < 0.001), and canagliflozin (100 mg, 15.00 ± 9.7%, *p* < 0.001) decreased the compulsive-like behaviors considerably by reducing the percentage of buried marbles when compared with the saline-treated VPA-exposed group. Interestingly, canagliflozin improved rats’ behavior during the marble-burying test in a dose-dependent manner, as a dose of 100 mg/kg of canagliflozin decreased the percentage of buried marbles substantially when compared with canagliflozin 20 mg/kg (*p* < 0.05).

#### 2.1.3. Nestlet-Shredding Test (NST)

The nestlet-shredding test was used to evaluate stereotyped- and compulsive-like behaviors of animals [33]. The observed results indicated that all treated VPA-exposed rats, including the ARP control group (3.52 ± 1.6%), canagliflozin group (20 mg) (3.38 ± 1%), canagliflozin group (50 mg) (1.99 ± 0.8%), and canagliflozin group (100 mg) (1.12 ± 0.6%) displayed a considerably reduced percentage of shredding behaviors compared with the no-treatment VPA-exposed group (12.99 ± 1.8%, *p* < 0.001). Moreover, a dose of 100 mg/kg of canagliflozin displayed remarkably (*p* < 0.01) compared with the reference drug ARP (3 mg) (Figure 1B).

### 2.2. Effects of Canagliflozin on ASD Biomarkers

#### 2.2.1. Oxidative Stress Biomarkers

**Figure 2:**
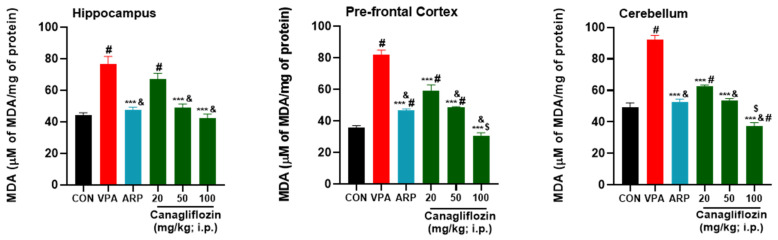
Canagliflozin and aripiprazole decrease oxidative stress biomarker (MDA) in the hippocampus, prefrontal cortex, and cerebellum. # *p* < 0.05 when compared with the CON group, & *p* < 0.05 when compared with the canagliflozin 20 mg group, $ *p* < 0.05 when compared with the ARP group, *** *p* < 0.001 when compared with the VPA group. Values are expressed as mean ± SEM (*n* = 6).

**Figure 3:**
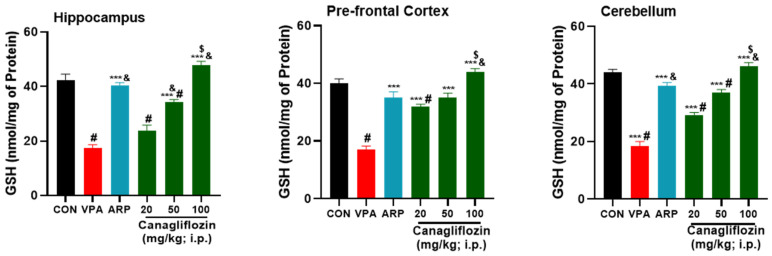
Canagliflozin and aripiprazole increase antioxidant (GSH) in the hippocampus, prefrontal cortex, and cerebellum. # *p* < 0.05 when compared with the CON group, & *p* < 0.05 when compared with the canagliflozin 20 mg group, $ *p* < 0.05 when compared with the ARP group, *** *p* < 0.001 when compared with the VPA group. Values are expressed as mean ± SEM (*n* = 6).

**Figure 4:**
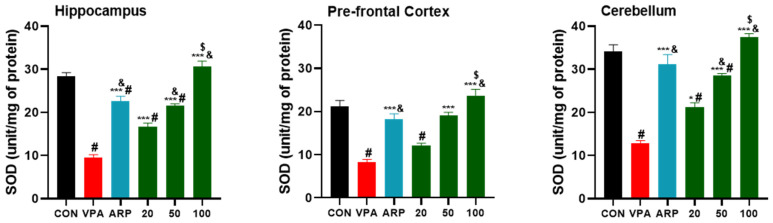
Canagliflozin and aripiprazole elevate antioxidant (SOD) in the hippocampus, prefrontal cortex, and cerebellum. # *p* < 0.05 when compared with the CON group, & *p* < 0.05 when compared with the canagliflozin 20 mg group, $ *p* < 0.05 when compared with the ARP group * *p* < 0.05 when compared with VPA group. *** *p* < 0.001 when compared with the VPA group. Values are expressed as mean ± SEM (*n* = 6).

**Figure 5:**
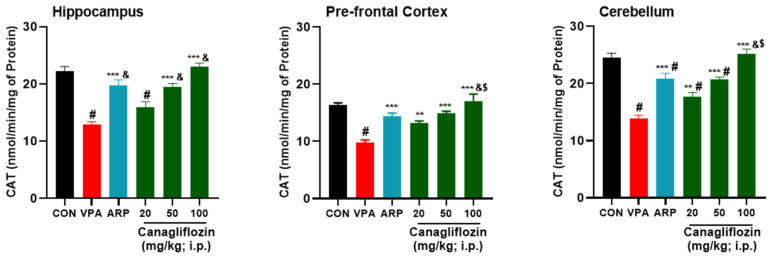
Canagliflozin and aripiprazole reduced neurotoxicity biomarker (AChE) levels in the hippocampus, prefrontal cortex, and cerebellum of VPA-exposed rats. # *p* < 0.05 when compared with the CON group, & *p* < 0.05 when compared with the canagliflozin 20 mg group, $ *p* < 0.05 when compared with the ARP group, ** *p* < 0.01 when compared with the VPA group, *** *p* < 0.001 when compared with the VPA group. Values are expressed as mean ± SEM (*n* = 6).

The results observed for the mitigating effects of the reference drug ARP (3 mg/kg) and canagliflozin (20–100 mg/kg) on the levels of oxidative stress tissues of the hippocampus, prefrontal cortex, and cerebellum are shown in Figure 2, Figure 3, Figure 4 and Figure 5. The observed results indicated that the modulating effects observed for canagliflozin (100 mg/kg) on malondialdehyde (MDA) levels were significantly higher than those witnessed for the reference drug ARP (3 mg/kg) in the prefrontal cortex and cerebellum (all *p* values < 0.001) (Figure 2). Moreover, the VPA-exposed group was found to have significantly superior levels of oxidative stress biomarker MDA (Figure 2) in comparison with control rats in all assessed brain regions (all *p* values < 0.001). However, the intraperitoneal administration of canagliflozin (20 mg/kg) significantly diminished the levels of MDA (*p* < 0.001) in the prefrontal cortex and cerebellum when compared with the VPA-exposed group (Figure 2) but failed to show a significant mitigating effect on MDA in hippocampal tissues of VPA-exposed animals (*p* > 0.05). Furthermore, the higher doses of canagliflozin (i.e., 50 and 100 mg/kg) significantly increased the lowered levels of glutathione (GSH), superoxide dismutase (SOD), and catalase (CAT) in each of the tested brain regions when compared with the saline-treated VPA-exposed animals’ group. The saline-treated VPA-exposed rats showed significantly increased levels of oxidative stress, expressed as significantly reduced levels of GSH, SOD, and CAT in the hippocampus, prefrontal cortex, and cerebellum (all *p* values < 0.001), when compared with control animals’ group. In addition, the observed results revealed that canagliflozin (100 mg/kg) significantly increased GSH levels, with mitigating effects significantly higher than those obtained for the reference drug ARP (3 mg/kg) in all assessed brain regions (all *p* values < 0.05) (Figure 3). 

The results indicated that SOD levels were significantly higher following systemic treatment with canagliflozin (100 mg/kg) than with ARP (3 mg/kg) (hippocampus, *p* < 0.001; prefrontal cortex and cerebellum, *p* < 0.05) (Figure 4). Overall, canagliflozin was able to decrease free radicals’ production and increase CAT activity in different brain regions of VPA-exposed rats in a dose-dependent manner as 100 mg/kg of canagliflozin displayed significantly higher ameliorative effects on CAT activity than 20 or 50 mg/kg of canagliflozin as well as the reference drug ARP (3 mg/kg) (Figure 5).

#### 2.2.2. Acetylcholinesterase (AChE) Activity

**Figure 6:**
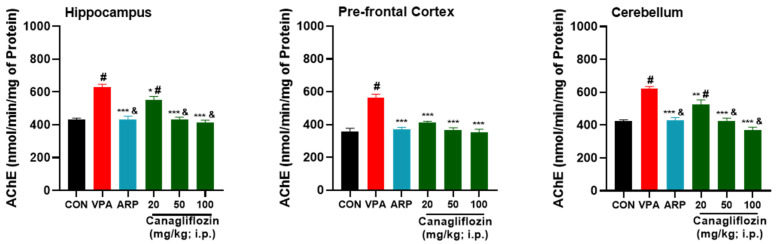
Canagliflozin and aripiprazole enhance antioxidant (CAT) in the hippocampus, prefrontal cortex, and cerebellum. # *p* < 0.05 when compared with the CON group, & *p* < 0.05 when compared with the canagliflozin 20 mg group, * *p* < 0.05 when compared with the VPA group, ** *p* < 0.01 when compared with the VPA group, *** *p* < 0.001 when compared with the VPA group. Values are expressed as mean ± SEM (*n* = 6).

Quantitative analysis has revealed a considerable increase (*p* < 0.001) in the AChE activity in the hippocampus, the prefrontal cortex, and the cerebellum of VPA-treated rats in comparison with the untreated control group (Figure 6). However, systemic subchronic treatment with canagliflozin (20 mg/kg) significantly reduced the AChE activity in the hippocampus and cerebellum (*p* < 0.05) as well as the prefrontal cortex (*p* < 0.001) of treated VPA-exposed rats compared to saline-treated VPA-exposed rats. Interestingly, the effects observed for canagliflozin on AChE activity were dose-dependent, as a higher dose of canagliflozin (50 mg/kg) could provide a significantly stronger reduction of AChE levels than those obtained with a dose of 20 mg/kg in the hippocampus (*p* < 0.001) and cerebellum (*p* < 0.05). Similarly, 100 mg/kg of canagliflozin resulted in more remarkable reduction (*p* < 0.001) of AChE activity in the hippocampus and cerebellum when compared with 20 mg/kg of canagliflozin, indicating a dose-dependent mitigating effect of canagliflozin on AChE activity (Figure 6).

### 2.3. Behavioral and ASD Biomarkers’ Response to Treatment Based on Gender

The observed results for behavioral and biochemical assessments were compared statistically to determine whether there are differences in the treatment response between male and female experimental animals (Table 2). Statistical analyses revealed that VPA-exposed male rats exhibited a considerably higher percentage (14.05 ± 1.97%, *p* = 0.011) of shredding behaviors in NST compared with female VPA-exposed rats (11.93 ± 0.11%). Similarly, VPA-exposed female rats treated with ARP showed a notably (2.21 ± 1.02%, *p* = 0.002) lower percentage of shredding behaviors compared to male VPA-exposed rats (4.82 ± 0.82%). However, the results observed in the NST, MBT, and OFT revealed that both genders responded equivalently well in their behaviors to 20, 50, and 100 mg/kg of canagliflozin. Regarding the results observed for oxidative stress levels in different specific brain regions, no statistically significant difference between either gender was shown in the hippocampus of treated VPA-exposed rats. However, the reference drug ARP (3 mg/kg) increased the GSH levels in the prefrontal cortex of female VPA-exposed rats (38.60 ± 3.47) significantly higher (*p <* 0.05) than those in the same region of male VPA-exposed rats (31.40 ± 3.61) (Table 2). Additionally, and in the prefrontal cortex, female VPA-exposed rats treated with 100 mg/kg of canagliflozin displayed significantly (18.49 ± 2.54, *p* < 0.05) higher levels of CAT compared to treated male VPA-exposed rats (15.63 ± 3.04). In contrast, the obtained results for MDA levels in the prefrontal cortex indicated that male VPA-exposed rats (53.42 ± 10.40) responded substantially better (*p* < 0.01) to a systemic treatment with 20 mg/kg of canagliflozin than female VPA-exposed rats (64.89 ± 1.17) (Table 2). Interestingly, the reference drug ARP (3 mg/kg) decreased the male cerebellar levels of SOD of treated rats to significantly lower (*p* < 0.05) than those obtained in cerebellar tissues of treated male rats, with 28.43 ± 6.96 and 33.80 ± 3.12, respectively (Table 2). Moreover, the results of statistical analyses revealed that the SOD levels in the prefrontal cortex of the control group were significantly higher (*p* < 0.05) in male VPA-exposed rats than those in females, with 23.60 ± 3.25 and 18.76 ± 4.40, respectively. Nevertheless, no significant differences between the two genders were witnessed for SOD levels in the prefrontal cortex and following systemic subchronic treatment with the reference drug ARP or the test compound canagliflozin (Table 2). 

## 3. Discussion 

Alteration in brain oxidative stress levels and AChE activity plays a crucial role in the pathogenesis of ASD-related behavioral features [34,35,36]. Several previous preclinical observations have shown that the SGLT2 inhibitor canagliflozin has displayed promising potential to mitigate oxidative stress and inflammation, in addition to its increasing effects on antioxidant capacity in numerous neurological disorders [15,16,17,22,27]. Therefore, the aim of the presented study was to evaluate the mitigating effects of canagliflozin on the oxidative stress levels in specific brain regions including the hippocampus, prefrontal cortex, and cerebellum of VPA-exposed rats with confirmed ASD-like behaviors.

Interestingly, Food and Drug Administration (FDA)-approved medications risperidone and aripiprazole for therapeutic management of ASD in children were found to reduce locomotor activity in different neurological disorders in in experimental animals with ASD-like behaviors [37], which is in agreement with our current results, as all doses of canagliflozin (20–100 mg/kg) significantly decreased locomotor activity of tested VPA-exposed rats in the OFT and in a pattern comparable to that observed for FDA-approved medications (Table 1). Notably, numerous previous preclinical studies indicated that antipsychotics such as risperidone reduced repetitive self-grooming in BTBR mouse models and Wistar rats’ ketamine model of schizophrenia [38,39]. Similarly, the observed results of this study revealed that canagliflozin at a higher dose (100 mg/kg) significantly reduced repetitive self-grooming behaviors of treated VPA-exposed rats, unlike other doses of canagliflozin (20 and 50 mg/kg) or the reference drug ARP (3 mg/kg), which failed to provide any appreciable ameliorating effects (Table 1).

The current observations showed that the various doses of canagliflozin ameliorated the stereotyped compulsive and repetitive-like behaviors in the MBT and NST of VPA-treated rats (Figure 1 and Figure 2). These observations were in line with previous preclinical studies conducted with several glucose-lowering agents in experimental rodents with ASD-like behaviors, including metformin [40].

According to previous preclinical observations, ARP was found to significantly reduce the elevated MDA levels in the hippocampus and cortex of rats with ketamine-induced schizophrenia [41]. In agreement with these results, our present observations for higher doses of canagliflozin (i.e., 50 and 100 mg/kg dose) as well as the reference drug ARP (3 mg/kg) showed a significant amelioration of MDA surge in the prefrontal cortex and cerebellum. However, the lower dose of canagliflozin (20 mg/kg) showed no significant difference in hippocampal MDA levels.

Similarly, our observations showed that canagliflozin (100 mg/kg) increased the reduced quantities of GSH in all tested brain regions (hippocampus, prefrontal cortex, and cerebellum), while canagliflozin (50 mg/kg) increased it in the prefrontal cortex and cerebellum, whereas a dose of 20 mg/kg managed to increase the GSH levels in only the prefrontal cortex. The latter observations for brain GSH levels are in harmony with previous preclinical observations for empagliflozin [42]. Interestingly, another preclinical study indicated that 2.5 mg/kg of ARP was capable of increasing GSH levels of Wistar rats with depression-like behaviors [43], which was comprehended with our present findings for ARP (3 mg/kg) and its mitigating effects on disturbed GSH levels of VPA-exposed rats. Furthermore, and as claimed by a previous animal study, canagliflozin (10 mg/kg) was found to boost cerebral cortex SOD levels and to provide neuroprotective effects in cisplatin-induced cerebral cortex injury of experimental rats [44]. In agreement with the latter observations, the current results comprehended that canagliflozin (20 mg/kg) increased SOD levels remarkably in the hippocampus and cerebellum of treated VPA-exposed animals, whereas higher doses of canagliflozin (50 and 100 mg/kg) cause a significant rise in SOD levels in all assessed brain regions (hippocampus, prefrontal cortex, and cerebellum). Interestingly, a plant called *Bacopa monnieri* (Brahmi) was found to ameliorate VPA-induced ASD-like behaviors in male rats due to their potent antioxidant benefits, as 300 mg/kg of *Bacopa monnieri* were found to elevate CAT activity and retrieved histoarchitecture of the cerebellum [45]. Our observations in the present study demonstrated similar enhancing effects on CAT activity in the prefrontal cortex and cerebellum following treatment of VPA-exposed rats with 20 mg/kg of canagliflozin, and in all three assessed brain regions when treated with higher doses of canagliflozin or ARP.

Notably, a previous preclinical experiment in rodents showed that canagliflozin (10 mg/kg) noticeably downregulated hippocampal AChE activity in scopolamine-induced memory impairment model in rats [46]. In line with these observations, our obtained results revealed that all doses of canagliflozin (20, 50, and 100 mg/kg) were able to significantly reduce AChE activity assessed in the hippocampus, prefrontal cortex, and cerebellum of VPA-exposed rats with ASD-like features. A recent study in postnatal VPA exposed rats (300 mg/kg) tested the ability of oral canagliflozin (5 mg/kg/ day, 7.5 mg/kg/day, and 10 mg/kg/day) in ameliorating autistic-like behaviors through the activation of phosphatase, tensin homolog (PTEN), pyruvate dehydrogenase kinase (PDK), and proliferator-activated receptor γ (PPAR γ) signaling pathways [47]. Results indicated that the treated group displayed neuroprotective features that were ascribed to the signaling pathways and also displayed increased sociability, as well as reduced stereotypic behavior and hyper-locomotor activity [47]. However, unlike the current study, they did not validate their autistic model and did not induce autism prenatally, as pups were injected with 300 mg/kg of VPA twice at PD-2, PD-3, and once at PD-4, following a protocol referenced by two studies [48,49]. In the current study, ASD induction was achieved by following a protocol validated in 149 studies [9] and the ASD model was validated by observing tail abnormalities. Moreover, Elgamal and colleagues [47] did not select a positive group treated with a compound that is approved for treating ASD, unlike the current study where ARP (3 mg/kg) was used to compare the effect of canagliflozin with an established ASD treatment molecule.

As stated in a previous study [50], and following our current observations, male VPA-exposed rats exhibited ASD-like behaviors, including repetitive/stereotyped-like behaviors and increased anxiety levels, whereas female VPA-exposed rats failed to display any appreciable alterations in these behaviors. Indeed, the observed results in the NST showed that male VPA-exposed rats exhibited more repetitive–compulsive behaviors than female VPA-exposed rats, except for repetitive self-rooming behaviors, locomotor activity, anxiety-like features, and behaviors assessed in MBT, where there was no significant difference between genders in the tested groups. However, the latter observed preclinical gender-based differences in behaviors were not translated into differences of treatment responses on the clinical level. In a study that included 585 male and 97 female participants (mean = 7.4 years; range 3–17 years) with ASD, measures of social disability, repetitive behavior, adaptive skills, disruptive behavior, and anxiety pretreatment were collected, followed by applying the improvement scale of the clinical global impression at the study endpoint, there were no appreciable gender-based differences in ASD treatment responses, since the rate of positive response was 53.6% in females and 49.7% in males [51]. Our present behavioral results clearly indicated that 50 and 100 mg/kg of canagliflozin exhibited positive results in both genders without significant difference between them. In fact, female VPA-exposed rats responded substantially better in the NST to 3 mg/kg of the reference drug ARP. In addition, ARP (3 mg/kg) provided significantly higher mitigating effects on the prefrontal cortical GSH levels, but significantly lower effects on the cerebellar SOD levels of female VPA-exposed rats. Moreover, in regard to CAT and MDA, both female and male VPA-exposed rats showed no significant differences in their responses to canagliflozin (50 mg/kg). however, at a higher dose of canagliflozin (100 mg/kg), cortical CAT and cerebellar MDA levels of female VPA-exposed rats were evidently more mitigated than those of male VPA-exposed rats. Interestingly, a lower dose of canagliflozin (20 mg/kg) substantially attenuated the cortical levels of MDA cortex in males than in females, whereas all subchronic systemic treatments with canagliflozin (20–100 mg/kg) provided significant mitigating effects on all oxidative stress markers in the hippocampus of treated VPA-exposed rats without witnessing any gender-based appreciable differences.

Up to our most recent knowledge, this study is the first in vivo illustration of the ameliorating effects of canagliflozin in comparison with ARP on various behavioral and biochemical characteristics in VPA-induced ASD in rats. Moreover, this study is the first of its kind to compare male and female VPA-exposed Wistar rats’ response to three different doses of canagliflozin compared to ARP. Furthermore, the fact that a non-diabetic rat model was used in this study confirms that canagliflozin potentially brought about these effects by mechanisms which are independent of their glucose-lowering action.

However, one of the main limitations of present study is the lack of assessment of the effects of canagliflozin on sociability and social novelty behavioral parameters of tested rats. Additionally, more neuroinflammatory biomarkers should be tested to determine the possible effects of canagliflozin on neuroinflammation in the VPA-exposed rats with ASD-like features.

## 4. Materials and Methods

### 4.1. Animals

The rats which were used in this study were bred in-house from the original stock which was purchased from Harlan Laboratories (Harlan Laboratories, Oxon, UK). The material of cages in which rats were housed was polypropylene plastic (dimensions: 43 × 22.5 × 20.5 cm^3^) under the following conditions: 12:12 h day/night cycle, in climate- and access-controlled rooms (23 ± 1 °C; 50 ± 4% humidity). Additionally, food and water were available ad libitum. The food was purchased from Emirates Feed Factory (Abu Dhabi, UAE), which is a standard maintenance diet for rats. For breeding, Wistar rats of both genders were housed together. To observe semen, an absorbent cage paper was placed under the cage. Gestational day 0 (GD-0) was counted when the vaginal plug was detected. Thereafter, rats that got pregnant were separated in different cages until delivery, which was defined as postnatal day 0 (PND-0). 

The entire experiment was held under the “Guiding Principles in the Care of and Use of Laboratory Animals”. Animals were handled, ethically treated, and humanly killed according to the rules and instructions of the Ethical Committee. All procedures that were carried out in accordance with the recommendations of the European Communities Council Directive of 24 November 1986 (86/609/EEC) were approved by the Institutional Animal Ethics Committee in the College of Medicine and Health Sciences, United Arab Emirates (Approval No. ERA-2017-5603). 

The number of animals selected was based initially using the resource equation method, without assuming the effect size. This method suggested the sample size of 30. However, the current study selected 36 animals to account for death or irregularity in results [52]. Confirmatively, after using the G*Power 3.1 software, measuring the effect size by the difference between the means of the control group and the treated group to understand the minimum difference that would be considered significant, the effect size was 1.03, which means practical finding has high significance, and a total sample size of 30 rats was assumed (6/group).

#### 4.1.1. Research Design

**Figure 7:**
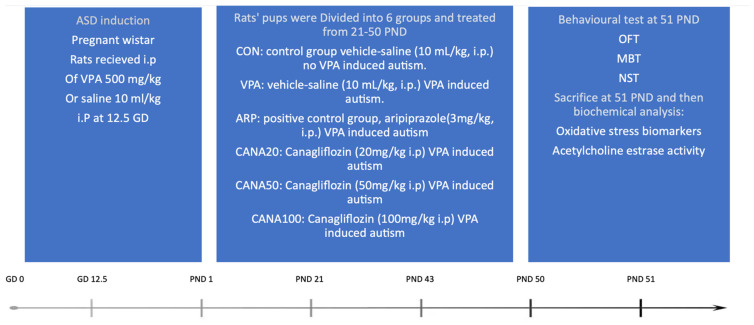
Graphical illustration of the implemented project plan, treatments, behavioral examinations, and biochemical analysis with autistic and control Wistar rats. At gestational day 12.5 (GD 12.5), pregnant rats were injected intraperitonially either with VPA (500 mg/kg) or saline (10 mL/kg). After delivery, starting from postnatal day 21, male and female pups were subdivided into 6 groups (6 rats/group) and received intraperitoneally (i.p.) the following treatments until PND 50, which is shown in the experimental design. Group 1: control rats injected with saline (10 mL/kg). Group 2: VPA-induced autism rats were injected with saline (10 mL/kg). Group 3: VPA-induced autism rats were injected with aripiprazole (3 mg/kg) and considered as the positive control. Group 4: VPA-induced autism rats were injected with 20 mg/kg of canagliflozin. Group 5: VPA-induced autistic rats were injected with 50 mg/kg of canagliflozin. Group 6: VPA-exposed autistic rats were injected with 100 mg/kg of canagliflozin. All 6 groups were subjected to behavioral assessment at PND 51 and scarified to undergo biochemical assays. OFT—open field test; MBT—marble-burying test; NST—nestlet-shredding test.

This research is an in vivo animal study to examine the efficacy of canagliflozin in VPA-induced autism in Wistar rats (Figure 7). In all the preformed tests, the observer was blinded to allocation of treatment groups.

#### 4.1.2. Choice of Treatment and Dosages

The dual-active canagliflozin was obtained from TRC (Toronto, Canada), and the rational for the dosing of compounds tested (20, 50, and 100 mg/kg i.p.) was based on previous studies [53,54,55]. Aripiprazole was purchased from Sigma-Aldrich (St. Louis, MO, USA), and the selected dose (3 mg/kg i.p.) was selected according to previously published studies [56,57]. VPA was also purchased from Sigma-Aldrich (St. Louis, MO, USA) and the choice of dosage for ASD induction (500 mg/kg i.p.) was based on a recent systematic review [9].

#### 4.1.3. Prenatal Treatment

Pregnant female rats were injected intraperitoneally (i.p.) with either VPA 500 mg/kg or saline 10 mL/kg on GD-12.5, after which they were returned to their home cages, as described previously. All pregnant rats stayed alive and no incidence of still birth was observed. Both female and male offspring were utilized in the study. Those who were delivered from VPA-treated mothers with one of 4 tail abnormalities including short tail, bent-in-the-middle tail, short-and-bent tail, as well as double-flexure tail were diagnosed with VPA-induced ASD [58] and allocated into VPA and treatment groups (group 2, 3, 4, 5, and 6). All obtained offspring were weaned, grouped by their gender, classified, and treated (3 rats/cage) at PND21. 

#### 4.1.4. Postnatal Treatment

On PND-21, offspring who were born to VPA-treated mothers and exhibited autistic features, as with those from mothers who received saline (control group), were divided into 6 subgroups (6 rats, 3 males and 3 females, per group) and received the following interventions by i.p. route as shown in experimental design (Figure 6). Group 1: control rats were injected with saline (10 mL/kg). Group 2: rats with VPA-induced ASD were injected with saline (10 mL/kg). Group 3: rats with VPA-induced ASD were injected with aripiprazole (3 mg/kg) and considered as the positive control. Group 4: rats with VPA-induced ASD were injected with 20 mg/kg of canagliflozin. Group 5: rats with VPA-induced ASD were injected with 50 mg/kg of canagliflozin. Group 6: rats with VPA-induced ASD were injected with 100 mg/kg of canagliflozin. All 6 groups were subjected to behavioral assessment at PND 51 and euthanized to undergo biochemical assays. Co-administered medications were given in separate injections with 5 min intervals following the injection of the test compound for 29 days, from PND 21 to 50. Treatment started at PND 21 as in human years, Wistar rats would be approximately 2 years old [59], which is considered as an effective early intervention according to a meta-analysis [60]. 

### 4.2. Behavioral Tests

#### 4.2.1. Open Field Test (OFT)

The open field test was originally developed as an experiment for emotionality evaluation in animals with neurodevelopmental disorders [61]. In this test, rats were randomly assigned and placed in a white acrylic square-form open-field arena of different dimensions (100 × 100 × 40 cm), following the previously published protocol [62]. Rats were placed at the center of the arena allowing them to explore the environment for 1 min in the beginning, then spontaneous activity measurement was applied for an hour. Rats were tested for spontaneous locomotor activity to assess emotionality [63], excessive and abnormal grooming patterns as a phenotype of repetitive and aggressive behavior [64], defecation, time that is spent in the central zone, time which was spent out of the central zone (periphery), and finally the total distance that was traveled, in order to ultimately measure fear and anxiety [31].

#### 4.2.2. Marble-Burying Test (MBT)

Odor-free, fresh bedding was added to all standard rat cages and leveled up to 5 cm to impress parallel lines that are suitable for marble placement. Four clean glass toy marbles were placed on the surface of each cage bedding with an order of 5 rows and a total of 20 marbles per cage.

Each rat was placed into the cage far from the marbles to avoid accidental burying, and the top cover was placed to prevent external disturbance. Rats remained in the cage for 30 min excluding food and water. The number of buried marbles was counted manually by observation. A marble was considered to have been buried if two-thirds of the marble surface area was under the bedding. The average score of buried marbles was used to assess the repetitive behavior [65].

#### 4.2.3. Nestlet-Shredding Test (NST)

The bedding was added to all standard rat cages, then leveled up to 0.5 cm. Cotton fiber nestlets were used for the NST and weighed initially then inserted into the cage bedding. Rats were placed separately into a cage with a single, pre-weighed nestlet, then the top cover was placed, excluding food and water for 30 min to avoid disturbance and external influence. The rats were taken away and returned to their original cages after the test was completed. The intact nestlet was removed and dried for 24 h at room temperature. To calculate the percentage of the shredded nestlet, the remaining un-shredded nestlets were weighed and their weight was divided by the baseline weight to provide an accurate and sensitive evaluation of repetitive and compulsive-like behaviors [66].

### 4.3. Biochemical Analyses

#### 4.3.1. Brain Extraction and Conditioning for Biochemical Assessments

After behavioral experiments, rats were anesthetized with pentobarbital (40 mg/kg, i.p.). Then, cardiac perfusion was achieved with 1× phosphate buffer saline (PBS) (composition: 0.01 M phosphate buffer, 0.0027 M potassium chloride, and 0.137 M sodium chloride) following a published protocol [67]. The hippocamps, prefrontal cortex, and the cerebellum were excised and rapidly frozen in liquid nitrogen for preservation to biochemical assays. The collected brain regions were later homogenized in accordance with published protocol [68], then supernatant was obtained for measuring the levels of GSH, MDA, SOD, CAT, and AChE.

#### 4.3.2. ASD Biomarkers Assessment Reagents and Experimental Kits

The lipid peroxidation assay kit for malondialdehyde evaluation (MDA, Lot no: MDA-2409, Product code: NWK-MDA01) was obtained from Northwest Life Science (Vancouver, WA, USA). The reduced glutathione assay kit (GSH Assay Kit, Lot no: 095M4114V, Product code: 1002170877) was purchased from Sigma-Aldrich (St. Louis, MO, USA). The assay kit for superoxide dismutase (SOD, Batch no: 0538703, Item: 706002) was obtained from Cayman Chemical (Ann Arbor, MI, USA). The assay kit for catalase (CAT, Batch no: 0539007, Item: 707002) was purchased from Cayman Chemical (Ann Arbor, MI, USA). The acetylcholinesterase activity colorimetric assay kit (Lot no: GR 3295454-2, Product: ab65345) was purchased from BioVision (Milpitas, CA, USA) to assess AChE activity. All kits were utilized following the manufacturers’ instructions.

### 4.4. Statistics

Data obtained in behavioral experiments and biochemical assessments were displayed as means ± SD. The data were checked for normality through examining sampling distribution (−1.4 to +1.4). The differences between treatment groups were compared using one-way analysis of variance (ANOVA) followed by the post hoc with Tukey’s test. Univariate pairwise comparison was used to compare between genders in each group. The software GraphPad Prism 5.01 for Windows (GraphPad Software, Inc., San Diego, CA, USA, www.graphpad.com) was utilized to carry out all statistical analyses. The null hypothesis asserts that canagliflozin-treated groups will show no improvements in comparison with the VPA-exposed group or the ARP group; however, if *p* values were less than 0.05, then it will be considered statistically significant, and hence the null hypothesis will be rejected.

## 5. Conclusions

The glucose-lowering agent, canagliflozin, provided alleviating effects of autistic-like attributes, including repetitive–compulsive and anxiety-like behaviors. Moreover, canagliflozin mitigated disturbed oxidative levels and AChE activity in the hippocampus, prefrontal cortex, and cerebellum of treated VPA-exposed rats. In addition, gender-based observations revealed that a major factor in repetitive behavior displayed in the nestlet-shredding test of VPA-exposed rats when no treatment was involved; however, treated VPA-exposed groups exhibited ameliorating effects regardless of the gender in all conducted behavioral paradigms. Furthermore, the results of all biochemical assessments indicated that gender did not influence the mitigating effects observed for canagliflozin. Additionally, the effects observed for 100 mg/kg dose of canagliflozin on oxidative stress markers MDA, GSH, SOD, and CAT were substantially better than those witnessed with the reference drug ARP (3 mg/kg).

Since 100 mg/kg i.p. of canagliflozin significantly ameliorated GSH and SOD levels in all tested regions, as well as substantially improving CAT and MDA in the PFC and cerebellum and reducing shredded nestlet notably in behavioral assessment when compared with 3 mg/kg i.p. of aripiprazole, the null hypothesis was rejected.

The overall observations of the present results suggest that further research on the ameliorating effects of canagliflozin in different ASD animal models is warranted to generalize its potential therapeutic effects to be used on patients diagnosed with ASD, as canagliflozin is an FDA-approved drug available in the market and is considered safe for children.

## Figures and Tables

**Table 1:** Canagliflozin and aripiprazole restored the abnormal anxiety and hyperactivity observed in VPA-exposed rats in the OFT.

Parameter	Control	VPA Group	ARP	Canagliflozin (20 mg) (Group 4)	Canagliflozin (50 mg) (Group 5)	Canagliflozin (100 mg) (Group 6)
Time spent at center (s)	14.8 ± 3.18	3.00 ± 0.89 ^#^	9.33 ± 4.41 ^#,^*	7.16 ± 2.31 ^#^	7.83 ± 1.72 ^#,^*	13.00 ± 3.09 *^,&^
Time spent at periphery (min)	9.45 ± 0.31	9.57 ± 0.01 ^#^	9.50 ± 0.04 *	9.52 ± 0.04 ^#^	9.52 ± 0.02 ^#^	9.47 ± 0.03 *
Locomotion (s)	147 ± 42.7	303 ± 140 ^#^	157 ± 41.6 *	161 ± 34.9 *	154 ± 44.7 *	147 ± 33.6 *
Distance traveled (m)	15.8 ± 5.37	27.9 ± 10.3 ^#^	17.3 ± 3.68 *	21.58 ± 6.06	20.0 ± 5.14	15.88 ± 1.59 *
Grooming (*n*)	5.33 ± 1.03	9.50 ± 2.88 ^#^	8.00 ± 3.03	9.83 ± 1.72 ^#^	7.17 ± 1.72	5.50 ± 0.83 *^,&^
Defecation (*n*)	3.33 ± 0.81	6.00 ± 1.78 ^#^	4.00 ± 1.41	5.00 ± 1.09	4.17 ± 1.72	3.83 ± 0.75

Values are expressed as mean ± SEM (*n* = 6). ^#^ *p* < 0.05 when compared with the CON group, ^&^ *p* < 0.05 when compared with the canagliflozin 20 mg group, * *p* < 0.05 when compared with the VPA group.

**Table 2:** Gender-based observations on behavioral features and oxidative stress levels of tested VPA-exposed rats.

Parameter/Group (Mean ± SD)	Control		*p*-Value	VPA		*p*-Value	ARP		*p*-Value	CANA20		*p*-Value	CANA50		*p*-Value	CANA100		*p*-Value
	*M*	*F*		*M*	*F*		*M*	*F*		*M*	*F*		*M*	*F*		*M*	*F*	
Time Spent at Center (s)	16.0 ± 4.3	13.6 ± 1.5	0.3	2.3 ± 0.5	3.6 ± 0.5	0.5	10.6 ± 2.5	8 ± 6.1	0.2	6.00 ± 1.00	8.3 ± 2.8	0.3	9 ± 1.7	6.6 ± 0.5	0.3	12.6 ± 3.7	13.0 ± 3	0.7
Time Spent at Periphery (min)	9.4 ± 0.04	9.4 ± 0.01	0.3	9.5 ± 0.01	9.5 ± 0.01	0.6	9.4 ± 0.02	9.5 ± 0.06	0.2	9 ± 0.01	9.5 ± 0.04	0.1	9.5 ± 0.01	9.5 ± 0.01	0.3	9.4 ± 0.03	9.4 ± 0.03	0.7
Locomotion (s)	160 ± 59.9	133.3± 21	0.6	337 ± 173	268 ± 123	0.2	158.3 ± 61.6	156 ± 23	0.9	137 ± 28.5	184.3 ± 22	0.4	143 ± 53.3	165 ± 42.2	0.7	151.6 ± 50.5	141.6 ± 13.5	0.8
Distance Traveled (m)	17.7 ± 7.5	13.9 ± 2.1	0.4	32.1 ± 13.0	23.6 ± 6.5	0.1	16.9 ± 5	17.7 ± 2.8	0.8	26.3 ± 3.50	16.8 ± 3.4	0.05	21.1 ± 6.7	18.8 ± 4.1	0.6	16.1 ± 1.6	15.7 ± 1.8	0.9
Grooming (mean ± SD)	4.6 ± 0.5	6 ± 1	0.4	9.00 ± 4.30	10 ± 1	0.5	8.6 ± 4	7.3 ± 2.3	0.4	10.3 ± 2.08	9.3 ± 1.5	0.5	7.3 ± 0.6	7 ± 2.6	0.8	5.3 ± 1.1	5.6 ± 0.5	0.8
Marble Buried (%)	18.3 ± 12.6	10 ± 5	0.2	55 ± 5	46.6 ± 2.8	0.2	25 ± 5	26.6 ± 14.4	0.7	31.6 ± 10.4	30 ± 5	0.7	21.6 ± 7.6	33.3 ± 2.8	0.08	15 ± 5	15 ± 8.6	1
Nestlet Shredded (%)	1.1± 0.2	1.1± 0.1	0.9	14.1 * ± 1.9	11.9 ± 1.03	0.011 *	4.83 ** ± 0.80	2.2 ± 1.02	0.002 **	3.7 ± 1.30	3 ± 0.7	0.3	2.5 ± 0.8	1.4 ± 0.2	0.1	1.5 ± 0.5	0.7 ± 0.5	0.2
MDA (μm MDA/mg of protein)																		
Hippocampus	46.6 ± 3.2	42.1 ± 1.6	0.4	81.0 ± 14.7	72.7 ± 6.7	0.1	50.9 ± 1.9	44 ± 3.7	0.2	67.6 ± 1.30	66.6 ± 14.1	0.86	53.4 ± 4.1	44.8 ± 1.4	0.1	45.9 ± 6.2	38.9 ± 4.8	0.2
Prefrontal Cortex	34.7 ± 1.9	36.8 ± 4.2	0.5	84.6 ± 4.10	79.8 ± 8.6	0.2	46 ± 2.8	47.4 ± 1.5	0.7	53.40 * ± 10.40	64.9 ± 1.10	0.006 **	47.7 ± 0.5	49.3 ± 1.8	0.6	33.3 ± 2.6	28.2 ± 4.6	0.1
Cerebellum	54.3 ± 5.30	44.5 ± 2.50	0.011 *	92.6 ± 7.70	92.3 ± 5.20	0.9	53.3 ± 4.6	51.4 ± 6	0.6	63.30 ± 2.4	61.6 ± 1.80	0.65	52.6 ± 3	54.1 ± 4.4	0.6	42.5 ** ± 2.30	31.7 ± 1.1	0.006 **
GSH (nmol/mg of protein)																		
Hippocampus	41.7 ± 2.8	43.2 ± 7.5	0.6	18 ± 4.3	16.8 ± 1.3	0.7	40.5 ± 3.4	40.6 ± 0.6	0.9	25.4 ± 6.9	22.2 ± 2.6	0.3	33.7 ± 1.2	34.9 ± 3.4	0.7	45 ± 1	50.7 ± 2.2	0.07
Prefrontal Cortex	38.8 ± 3.9	41.2 ± 4.0	0.4	16.8 ± 2.1	17.2 ± 4.2	0.8	31.4 ± 3.6	38.6 ± 3.4	0.019 *	31.6 ± 1.2	32.2 ± 2.8	0.8	34.3 ± 4.5	35.7 ± 4.1	0.6	44.3 ± 3.1	43.6 ± 3.2	0.8
Cerebellum	43.6 ± 1.8	44.6 ± 3.1	0.6	17.4 ± 1.3	19.3 ± 5.8	0.4	40.7 ± 2.1	38.1 ± 2.8	0.3	28 ± 2.1	30.4 ± 1.5	0.3	37.8 ± 3.7	35.9 ± 1.8	0.4	46.3 ± 3.2	45.8 ± 4	0.8
GSH (nmol/mg of protein)																		
Hippocampus	41.7 ± 2.8	43.2 ± 7.5	0.6	18 ± 4.3	16.8 ± 1.3	0.7	40.5 ± 3.4	40.6 ± 0.6	0.9	25.4 ± 6.9	22.2 ± 2.6	0.3	33.7 ± 1.2	34.9 ± 3.4	0.7	45 ± 1	50.7 ± 2.2	0.07
Prefrontal Cortex	38.8 ± 3.9	41.2 ± 4	0.4	16.8 ± 2.1	17.2 ± 4.2	0.8	31.4 ± 3.6	38.6 ± 3.4	0.01 *	31.6 ± 1.2	32.2 ± 2.8	0.8	34.3 ± 4.5	35.7 ± 4.1	0.6	44 ± 3	43.6 ± 3.2	0.8
Cerebellum	43.6 ± 1.8	44.6 ± 3.1	0.6	17.4 ± 1.3	19.3 ± 5.8	0.4	40.7 ± 2.1	38.1 ± 2.8	0.3	28 ± 2.1	30.4 ± 1.5	0.3	37.8 ± 3.7	35.9 ± 1.8	0.4	46.3 ± 3.2	45.8 ± 4	0.8
SOD (unit/mg of protein)																		
Hippocampus	28.2 ± 3.6	28.3 ± 0.5	0.9	9 ± 2.1	10.1 ± 0.4	0.546	21.1 ± 2.8	24.2 ± 1.8	0.1	15.8 ± 1.8	17.4 ± 2.8	0.4	20.7 ± 1	22.2 ± 0.8	0.4	30.5 ± 2.1	30.7 ± 4.3	0.9
Prefrontal Cortex	23.6 ± 3.2	18.7 ± 0.8	0.03 *	8.6 ± 1.1	8 ± 1.8	0.772	17.9 ± 1.4	18.6 ± 4.4	0.7	12.1 ± 1.8	11.9 ± 1.4	0.9	18.4 ± 2.6	19.6 ± 1.4	0.5	23.5 ± 1.2	23.8 ± 5.4	0.9
Cerebellum	35.8 ± 4	32.4 ± 3.2	0.2	11.8 ± 2	13.6 ± 1.3	0.473	28.4 ± 6.9	33.8 ± 3.1	0.04 *	20.4 ± 1.9	21.9 ± 3.3	0.5	27.9 ± 0.9	29 ± 1.5	0.6	37.5 ± 1.4	37.4 ± 2.5	0.9
CAT (nmol/min/mg of protein)																		
Hippocampus	20.9 ± 1.9	23.4 ± 1.1	0.121	12.4 ± 1.3	13.3 ± 1.2	0.5	20.6 ± 3.2	18.8 ± 1.3	0.2	15.5 ± 2.9	16.3 ± 2.1	0.6	19.5 ± 1.9	19.4 ± 1.1	0.9	22.4 ± 0.6	23.6 ± 1.8	0.4
Prefrontal Cortex	16 ± 0.8	16.7 ± 0.5	0.586	9.8 ± 0.6	9.7 ± 1.5	0.9	13.4 ± 0.4	15.3 ± 1.2	0.1	13.1 ± 0.9	13.3 ± 0.9	0.7	14.8 ± 1.1	14.9 ± 0.4	0.9	15.6 ± 3	18.4 ± 2.5	0.02 *
Cerebellum	23.3 ± 1.6	25.5 ± 1.9	0.129	13.6 ± 1.7	14 ± 1.8	0.7	20.7 ± 1.3	21 ± 3.3	0.8	16.9 ± 1.5	18.5 ± 0.6	0.2	19.9 ± 0.4	21.4 ± 1.6	0.2	23.5 ± 0.9	26.8 ± 1.3	0.02 *
AChE (nmol/min/mg of protein)																		
Hippocampus	428.6 ± 36.1	431.6 ± 20	0.933	638.3 ± 48	622.6 ± 44.4	0.664	450 ± 41.1	419 ± 46.5	0.392	544.6 ± 48.6	557 ± 67.9	0.7	433.3 ± 47.3	428 ± 43.4	0.8	427.3 ± 36.1	404.3 ± 22.5	0.5
Prefrontal Cortex	382 ± 9.1	334.3 ± 59.7	0.125	545.3 ± 68.8	584.3 ± 17.5	0.205	387.3 ± 37.1	351 ± 20.8	0.237	425.3 ± 5.8	399.3 ± 22.3	0.3	345.3 ± 22.5	387.3 ± 34.7	0.1	379.6 ± 13.4	326 ± 56.6	0.08
Cerebellum	425.6 ± 25.8	423 ± 1	0.944	630 ± 45.5	612.6 ± 26.5	0.647	452.3 ± 27.7	408.3 ± 40.5	0.251	520.3 ± 65.2	530.6 ± 86.6	0.7	439.6 ± 10.6	413.6 ± 54.5	0.4	341.6 ± 38.1	393.6 ± 52.6	0.1

Values are expressed as mean ± SEM (*n* = 6). * *p* < 0.05, ** *p* < 0.01. *M*—Male. *F*—Female.

## Data Availability

The data of this study are available upon request.

## Abbreviations

AChE	Acetylcholinesterase
ADA	American Diabetes Association
AD	Alzheimer’s disease
ARP	Aripiprazole
ASD	Autism spectrum disorder
CAT	Catalase
CNS	Central nervous system
ESC	European Society of Cardiology
FDA	Food and Drug Administration
GD	Gestational day
GSH	Glutathione
HF	Heart failure
i.p	Intraperitoneally
MDA	Malondialdehyde
PD	Parkinson’s disease
PND	Postnatal day
SGLT 1	Sodium-glucose cotransporter 1
SGLT 2	Sodium-glucose cotransporter 2
SOD	Superoxide dismutase
T2DM	Type 2 diabetes mellitus
VPA	Valproic acid
